# Spermidine supplementation in rare translation-associated disorders

**DOI:** 10.15698/cst2021.03.243

**Published:** 2021-03-08

**Authors:** Andreas Zimmermann, Didac Carmona-Gutierrez, Frank Madeo

**Affiliations:** 1 Institute of Molecular Biosciences, NAWI Graz, University of Graz, Graz, Austria.; 2 Field of Excellence BioHealth – University of Graz, Graz, Austria.; 3 BioTechMed-Graz, Graz, Austria.

**Keywords:** eIF5A, hypusination, translation, rare disease, spermidine, Kabuki syndrome

## Abstract

The polyamine spermidine is essential for protein translation in eukaryotes, both as a substrate for the hypusination of the translation initiation factor eIF5A as well as general translational fidelity. Dwindling spermidine levels during aging have been implicated in reduced immune cell function through insufficient eIF5A hypusination, which can be restored by external supplementation. Recent findings characterize a group of novel Mendelian disorders linked to *EIF5A* missense and nonsense variants that cause protein translation defects. In model organisms that recapitulate these mutations, spermidine supplementation was able to alleviate at least some of the concomitant protein translation defects. Here, we discuss the role of spermidine in protein translation and possible therapeutic avenues for translation-associated disorders.

Spermidine is a naturally occurring polyamine that is strongly involved in manifold cellular processes, for example, DNA, RNA and protein stabilization, cell growth, proliferation, autophagy, and aging. Importantly, spermidine has also been connected to the modulation of general protein translation, and specifically also to the control of the translation factor eIF5A. This translation initiation factor features a unique amino acid derivative called hypusine, which is synthesized by the transfer of the 4-amino butyl moiety of spermidine to the ε-amino group of lysine 50 through the enzymes deoxyhypusine synthase and deoxyhypusine hydroxylase [1]. As this modification is essential for normal eIF5A function, insufficient hypusination results in dysfunctional translation of eIF5A targets, in particular proteins that contain polyproline tract (PPT) motifs. In detail, hypusinated eIF5A resolves ribosomal stalling that occurs at PPT motifs by entering the ribosomal E site when a diproline peptide is bound to the tRNA at the ribosomal P-site. The hypusine residue directly interacts with the acceptor stem of the P-site tRNA and reorients it to overcome the sterical restrictions imposed by the imino acid proline [2]. In addition, eIF5A can promote general translation in an hypusination-independent manner [3]. Still, eIF5A hypusination deficiency has been associated to health decline. For instance, accumulating evidence suggests that decreasing eIF5A hypusination may account for aging-associated defects in both B cell and T cell function through insufficient translation of lysosomal function-associated and mitochondrial proteins, respectively [4, 5].

In a recent study, Faundes *et al.* demonstrate that spermidine supplementation partially improves disorders associated to mutations in eIF5A in model organisms [6]. The authors identified a novel set of human Kabuki syndrome-like Mendelian disorders that phenotypically manifest in facial dysmorphism, microcephaly and micrognathia. On the molecular level, the disorders are characterized by an accumulation of PPT proteins linked to nonsense or missense *EIF5A* variants [6]. The missense variants affect amino acids at the surface of the protein, and one mutation in particular, T48N, is very close to the hypusinated residue lysine 50. To investigate the molecular consequences of the variants, the authors recapitulated the corresponding mutations in baker's yeast by expressing codon-optimized variants of the human gene. Expressing the wild type variant of human eIF5A did not affect growth rates, while expressing the disorder variants resulted in drastically impaired growth and loss of normal polysome profiles, an indicator for dysfunctional translation. In addition, the authors detected reduced eIF5A hypusination levels, which, however, was only consistently observed in the T48N variant.

The observed growth impairment is not surprising, as in yeast, eIF5A is not exclusively required for the resolution of PPT-associated ribosomal stalling, but is broadly involved in general translation elongation and termination [7]. Moreover, eIF5A hypusination is the main limiting factor for yeast growth under polyamine depletion [8]. Thus, any introduced disturbance of eIF5A function likely results in reduced cellular growth rates. While general translational capacity was not investigated, reporter gene expression of PPT-containing proteins confirmed that eIF5A variants reduced the cellular ability to synthesize at least some PPT-tract proteins [6].

Aging-associated impairment of eIF5A hypusination can be alleviated by supplementation of spermidine in mammalian cells and *in vivo*. In fact, declining spermidine levels during aging are likely to be causally linked to insufficient hypusination reactions [4, 5]. Moreover, polyamines are generally implicated in eukaryotic translation efficiency, for instance by lowering the need for Mg^2+^ ions, at least *in vitro* (**Figure 1**) [3, 9]. Accordingly, Faundes *et al.* tested, whether spermidine supplementation was able to alleviate dysfunctional translation in the humanized yeast model expressing the human disease associated mutations. Indeed, spermidine supplementation partially restored growth and polysome profiles. Interestingly, hypusination levels were not restored, suggesting that the introduced mutations either did not directly interfere with the hypusination reaction, or in the case of T48N, precluded efficient hypusination altogether, irrespective of the substrate (i.e., spermidine availability). This notion was further corroborated by experiments in zebrafish, where spermidine supplementation was able to reverse eif5a knockdown-induced micrognathia, a scenario where spermidine levels are unlikely to become rate-limiting for hypusination [6].

**Figure 1:**
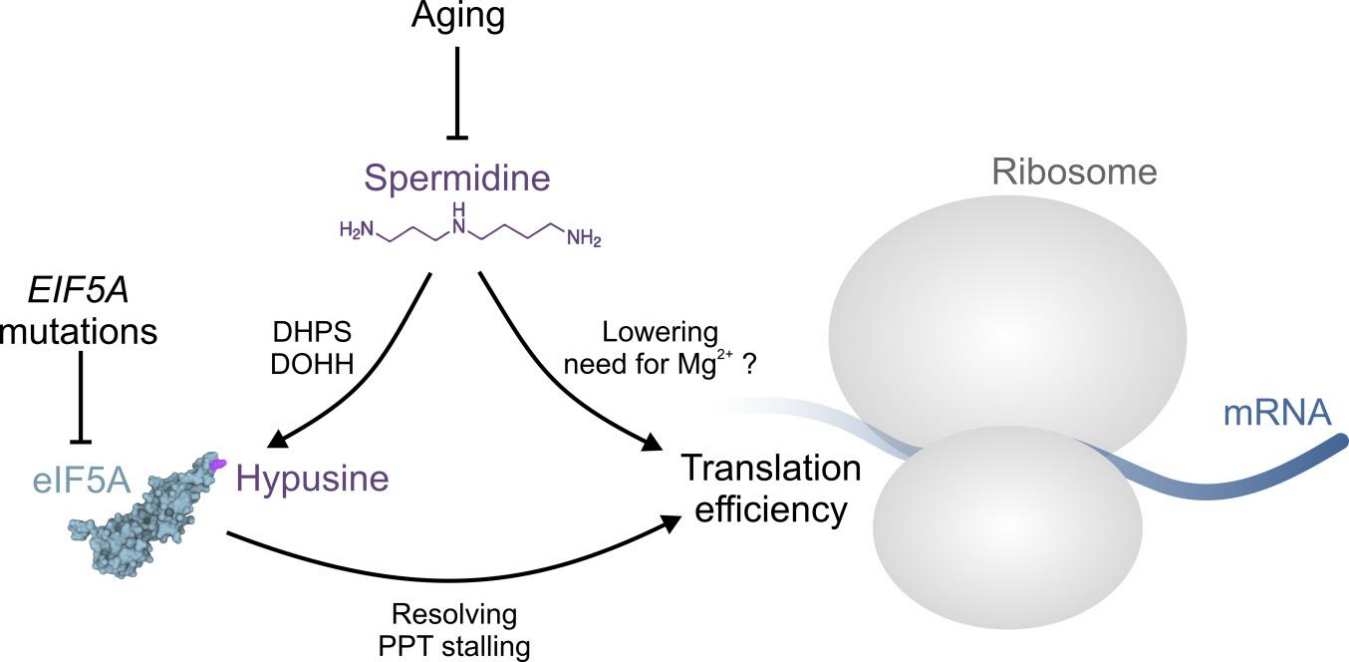
FIGURE 1: The role of spermidine in protein translation. Aging or *EIF5A* mutations can interfere with normal protein translation through insufficient eIF5A hypusination and/or function, respectively. Aging is accompanied by a decrease of available spermidine, which is required for the hypusination of eIF5A through deoxyhypusine synthase (DHPS) and deoxyhypusine hydroxylase (DOHH). Hypusinated eIF5A is able to resolve ribosomal stalling at poly-proline tract (PPT) motifs. Spermidine may promote translation hrough eIF5A-independent mechanisms, e.g. by lowering the need for Mg^2+^ ions. See text for details. The structure of eIF5A was generated based on the RCSB PDB (rcsb.org) entry 3ER0 representing the crystal structure of yeast eIF5A [19].

Protein translation-related defects are implicated in a wide array of (mostly rare) diseases [10], but also include the declining function of the immune system during aging [4, 5]. The role of spermidine as a donor for eIF5A hypusination as well as in general translation puts it in the spotlight as a potential therapeutic against disorders associated to translation defects. However, several limitations and unanswered questions should be considered: (i) With respect to eIF5A hypusination levels, it is not clear - from an enzyme kinetics perspective - if the typically measured cellular concentrations of spermidine can become rate-limiting at any point. It also remains unclear, which cellular pool of spermidine is able to fuel hypusination. Given the fact that only a fraction of polyamines is believed to be available as free, unbound molecules, which is likely the state they are required in for enzymatic reactions, it is tempting to speculate that the available fraction of spermidine for hypusination reactions might decrease at a faster rate than the total cellular polyamine content. Along those lines, data from yeast indicate that under spermidine-depleted growth conditions, about 50% of the remaining spermidine feeds into hypusination reactions [8], suggesting that there is a constant need for maintaining an eIF5A hypusination status. (ii) With respect to disorders specifically linked to eIF5A mutations, they might be insusceptible to spermidine supplementation, at least regarding hypusination, as demonstrated by Faundes *et al.* Similarly, it is unlikely that any intervention can revert developmental, prenatal defects caused by defective protein translation. In that light, more experimental work in appropriate animal models for the newly characterized disorders are required to determine the efficacy and required dose of spermidine.

What are the molecular mechanisms behind the beneficial effects of spermidine in protein translation? In *Escherichia coli*, an estimated 15% of polyamines are bound to ribosomes and as much as 90% of total polyamines are believed to be associated to nucleic acids, including cellular RNAs [11, 12], suggesting that they participate in protein translation at multiple steps. Polyamines, in particular spermidine and spermine, enhance translation efficiency in cell-free systems, an effect that is not necessarily dependent on the specific mRNA [13]. At the same time, polyamine levels can control the translation of specific mRNAs coding for proteins of the polyamine synthesis pathway as part of an auto-regulatory feedback loop [11]. When it comes to eIF5A-related translational activity, the role of polyamines is less understood, except for supplying 4-amino butyl groups for hypusination. Given the fact that eIF5A and spermidine can replace each other at the ribosomal P-site in some scenarios [3], it is possible that under conditions where eIF5A does not function properly, spermidine (and possibly other polyamines) may step in to take over some of the functions of eIF5A. Alternatively, dysfunctional eIF5A might affect cellular polyamine content, causing a reduction of general translational efficiency, which might be alleviated by external supplementation.

Dietary spermidine supplementation in humans seems safe [14], and supplementation in different model organisms even results in improved lifespan and healthspan [15, 16]. Consistently, nutritional spermidine intake inversely correlates with mortality in humans [17]. Besides its role in protein translation, spermidine also acts at the other end of a protein's life cycle, namely as an inducer of autophagy, an intracellular degradation pathway for macromolecules and even whole organelles [18]. Autophagy is required for many health outcomes of spermidine supplementation, suggesting that it is a major downstream effector [18]. Intriguingly, data from spermidine-depleted B-cells suggest that there might be a direct chain of events connecting spermidine's capacity to promote translation and autophagy induction: reduced cellular spermidine content reduces hypusination which, consequently, leads to diminished translation of the transcription factor TFEB, a PPT protein, which promotes the expression of lysosomal and autophagy-related genes [5]. External spermidine administration in turn enhances hypusination and TFEB mediated autophagy.

Apparently, the aging-related decline of spermidine levels can affect normal cellular function at multiple points. The growing number of indications, which can be treated by spermidine supplementation, at least in animal models, underscores the importance to further investigate the molecular mechanisms of spermidine-associated health benefits.
